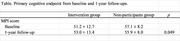# Exercise program to reduce the risk of cognitive decline in older adults

**DOI:** 10.1002/alz70860_101470

**Published:** 2025-12-23

**Authors:** Moeko Shinohara, Kunihiko Yokoyama, Junji Komatsu, Kazumi Mauda, Mitsunobu Kono, Mitsuhiro Yoshita, Kenjiro Ono

**Affiliations:** ^1^ Department of Neurology, Kanazawa University Graduate School of Medical Sciences, Kanazawa, Ishikawa, Japan; ^2^ Public Central Hospital of Matto Ishikawa, Hakusan, Japan; ^3^ Kanazawa University Graduate School of Medical Sciences, Kanazawa, Japan; ^4^ Kanazawa University, Kanazawa, Ishikawa, Japan; ^5^ Kinjo University, Hakusan, Ishikawa, Japan; ^6^ Hokuriku National Hospital, Nanto, Japan; ^7^ Kanazawa University Graduate School of Medical Sciences, Kanazawa, Ishikawa, Japan

## Abstract

**Background:**

We examined the efficacy of an intervention (physical exercise program and nutritional lectures) in preventing cognitive decline among older adults without dementia.

**Method:**

This study included non‐demented individuals aged 65 years and over. Intervention group received physical exercise training program once a week and nutritional lectures once a month for five months. Seventy‐six and 36 individuals completed baseline and 1‐year cognitive assessment using MCI screen in the intervention group and non‐participants group, respectively. The primary endpoint was the memory performance scores of the MCI screen.

**Result:**

The MCI screen score was found to be increased by 1.8 and decreased by 1.2 in the intervention and non‐participants groups, respectively; this difference was statistically significant (*p* = 0.049).

**Conclusion:**

These results indicate that a 5‐month intervention (physical exercise training program and nutritional lectures) for older adults without dementia could improve their cognitive function.